# Integrated hormone and transcriptome profiles provide insight into the pericarp differential development mechanism between Mandarin ‘Shatangju’ and ‘Chunhongtangju’

**DOI:** 10.3389/fpls.2024.1461316

**Published:** 2024-10-10

**Authors:** Yongjing Huang, Congyi Zhu, Yibo Hu, Sanjiao Yan, Zhimin Luo, Yanping Zou, Wen Wu, Jiwu Zeng

**Affiliations:** ^1^ Institute of Fruit Tree Research, Guangdong Academy of Agricultural Sciences, Key Laboratory of South Subtropical Fruit Biology and Genetic Resource Utilization, Ministry of Agriculture and Rural Affairs, Guangdong Provincial Key Laboratory of Science and Technology Research on Fruit Trees, Guangzhou, China; ^2^ Deqing County Agricultural Technology Promotion Center, Zhaoqing, China; ^3^ Longmen County Agricultural and Rural Comprehensive Service Center, Huizhou, China

**Keywords:** Mandarin ‘Shatangju’, Mandarin ‘Chunhongtangju’, pericarp, transcriptome, hormone

## Abstract

**Introduction:**

*Citrus reticulata* cv. ‘Chunhongtangju’ was mutated from Mandarin ‘Shatangju’, which has been identified as a new citrus variety. Mandarin ‘Chunhongtangju’ fruits were late-ripening for about two months than Mandarin ‘Shatangju’.

**Methods:**

To understand the pericarp differential development mechanism in Mandarin ‘Shatangju’ (CK) and ‘Chunhongtangju’ (LM), hormones and transcriptome profiles of pericarps were performed in different development stages: Young fruit stage (CK1/LM1), Expansion and Turning color stage (CK2), Expansion stage (LM2), Turning color stage (LM3), and Maturity stage (CK3/LM4).

**Results:**

In this study, the development of LM was significantly slower, and the maturity was significantly delayed. At the same stage, most hormones in Mandarin ‘Chunhongtangju’ pericarps were higher than that in ‘Shatangju’ such as gibberellin A24, cis(+)-12-oxophytodienoic acid, and L-phenylalanine. The deficiency of hormones in late-maturing pericarps was mainly manifested in ABA, 12-OHJA, MeSAG, and ABA-GE. Differences in transcriptome profiles between the two citrus varieties are primarily observed in energy metabolism, signal transduction such as MAPK signaling pathway and plant hormone signaling, and biosynthesis of secondary metabolites. After analyzing the hormones and transcriptome data, we found that the top genes and hormones, such as Cs_ont_5g020040 (transcription elongation factor, *TFIIS*), Cs_ont_7g021670 (BAG family molecular chaperone regulator 5, *BAG5*), Cs_ont_2g025760 (40S ribosomal protein S27, *Rps27*), 5-deoxystrigol, salicylic acid 2-O-β-glucosid, and gibberellin A24, contributed significantly to gene transcription and hormone synthesis.

**Discussion:**

This study suggests that the variances of pericarp development between the two varieties are linked to variations in the transcription levels of genes associated with energy and secondary metabolism, signal transduction related genes. These findings expand our understanding of the complex transcriptional and hormonal regulatory hierarchy during pericarp development.

## 1 Introduction

Citrus is widely cultivated around the world. Mandarin ‘Shatangju’ (*Citrus reticulata* cv.) is a unique citrus variety in Guangdong Province, China. Mandarin fruits have many advantageous characteristics including smell, thin exocarp, and enriched second metabolites (Zhang et al., 2012; Yu et al., 2018; Barry et al., 2020). The development of citrus fruits involves numerous biochemical and physiological changes within the fruit such as sugar accumulation and organic acid degradation (Lin et al., 2015). During the expansion stage, the acidity increases and then declines, and total soluble sugars (TSS) increase after the expansion stage (Carvalho et al., 2020; Silveira et al., 2020). The development of fruits is related to a variety of biotic and abiotic stress factors (Barry et al., 2020), such as hormones (Salehin et al., 2019), rootstock (Hernández et al., 2023), climate (Luo et al., 2023), and so on. The development and maturation of citrus fruits are also mainly by genetic factors (Shimada et al., 2005). Stiff person syndrome, glutamic acid decarboxylase, aspartate transferase, and ATP-citrate lyase genes contribute to fruit maturation (Lin et al., 2015).

The color transformation of citrus fruit pericarps from green to yellow or red is caused by changes in pigment composition and concentration, including chlorophylls and carotenoids (Conesa et al., 2019). Citrus pericarps were a complex source of carotenoids (Wang et al., 2008). Chlorophyll degradation and carotenoid biosynthesis are important processes in citrus fruit development and other metabolisms involved in fruit development (Sun et al., 2020). The pericarp tissue contains a variety of carotenoids, which contribute to the wide range of colors in citrus fruits (Sun et al., 2020). The characteristic color of most citrus fruits is mainly due to the accumulation of (9Z)-violaxanthin and β-cryptoxanthin (Iglesias et al., 2007). The metabolic signal regulatory network controls the color break during citrus fruit development, including ripening inducers and retardants such as ethylene, sucrose, gibberellins, and nitrogen (Alós et al., 2006). β-Limonin is a red pigment in the citrus pericarps of clementine and sweet orange. Its biosynthesis is regulated by the carotenoid lytic dioxygenase (*CitCCD4*) gene (Bilal et al., 2013; Wurtzel, 2013). Due to complex regulatory networks, The color transformation of the ‘*Verna*’ lemon is slower than the ‘*Fino*’ variety (Conesa et al., 2019). ‘*Eureka Frost*’, ‘*Lisbon Frost*’, and ‘*Fino 49*’ varieties start to carry out the color transformation two months earlier than the ‘*Verna*’ variety (Porras et al., 2010).

The development and maturation of plant fruits is a complex biological process that involves several intricate biological processes. These processes include the formation of flowers, pigments, aroma, sugar accumulation, and acid degradation and require the precise expression of numerous structural genes and transcriptional regulatory genes (Giovannoni, 2004; Jin et al., 2014). For example, the MADS-box family plays a crucial part in regulating the development of flowers (Theissen and Melzer, 2007), roots (Ku et al., 2008), and fruit (Bemer et al., 2012; Shima et al., 2014). The MYB transcription factor family is deeply involved in regulating plant secondary metabolism, hormone response, and pigment synthesis (Lea et al., 2007; Medina-Puche et al., 2014). *MaNACs* can interact with ethylene insensitive 3 (*EIN3*) and contribute to banana fruit ripening (Shan et al., 2012). Plant hormones regulate the growth and development of roots (Xie et al., 2021), leaves (Liu et al., 2022), flowers (Chandler, 2011), and so on. In particular, gibberellic acid (GA) can promote cell elongation (Kumar et al., 2014), while ethylene stimulates the development and ripening of non-climacteric fruits (Gapper et al., 2013; Osorio et al., 2013). Studies have revealed that GA3 does not delay citrus fruit coloration, but ethylene can accelerate degreening (Zacarias et al., 1998). Thus, the interplay between transcription factors and hormones is important in the development and maturation of citrus fruits. However, despite having similar genetic backgrounds, the differential developmental mechanisms of citrus fruits between the two citrus varieties, Mandarin ‘Shatangju’, and Mandarin ‘Chunhongtangju’, remain unclear.

Omics is a powerful tool used to analyze complex regulatory networks in various situations such as between varieties, processing, and tissues (Wang et al., 2015; Zhong et al., 2015; Wang et al., 2017). RNA-seq has been widely used to reveal various regulatory mechanisms and identify genes for different functions in citrus (Yu et al., 2012; Zheng et al., 2012; Aritua et al., 2013; Wang et al., 2015; Bi et al., 2022; Chen et al., 2022). In this study, the transcriptome was used to systematically analyze the different expression patterns of genes between two citrus varieties and during the development and ripening process of Mandarin ‘Shatangju’ and ‘Chunhongtangju’ fruits. Key differentially expressed genes were screened to explore the different mechanisms of fruit pericarps between the two citrus varieties during fruit ripening. Hormones play a crucial role in fruit development and ripening. Comprehensive research of transcriptome and hormone analysis can reveal key metabolism-related gene expression patterns of different citrus cultivars and key hormones for developing citrus fruits. Therefore, they would indicate the key genes and hormones that contribute to the development of citrus fruits.

In this research, the pericarps of citrus fruits were collected and analyzed to study the gene transcription patterns and hormone levels of two citrus varieties during different stages of fruit development. The study was conducted using transcriptome and UPLC-MS/MS techniques. This research aims to provide new insights into the molecular mechanisms that regulate fruit development in two closely related citrus varieties, namely Mandarin ‘Shatangju’ and ‘Chunhongtangju’.

## 2 Materials and methods

### 2.1 Plant materials

Two citrus varieties, namely *Citrus reticulata* cv. ‘Shatangju’ and ‘Chunhongtangju’, were selected for this study. The pericarp samples of both varieties were obtained from the citrus research orchard at the Institute of Fruit Tree Research, GDAAS, China (E 113°21’59’, N 23°9’15’). The fresh fruits were collected every two months from August 2022 to February 2023. Mandarin ‘Shatangju’ fruits were classified into three stages, named ‘Control_1/2/3’ (CK1/2/3) according to the development stages A. Whereas, ‘Chunhongtangju’ were classified into four stages, named ‘Late-maturing_1/2/3/4’ (LM1/2/3/4). The CK1/2/3 samples were collected at 220, 250, and 280 days after flowering (DAF), and the LM1/2/3/4 samples were collected at 220, 250, 280, and 310 DAF. The colorimeter (Minolta CR-300, Konica Minolta Investment Ltd, Shanghai, China) was used for detecting the coloration of citrus pericarps. Three biological replicates were set for each sample containing 30 fruits randomly collected from twenty Mandarin ‘Shatangju’ or ‘Chunhongtangju’. The pericarp was excised with scalpels, frozen in liquid nitrogen, and kept at ultra-low temperature.

### 2.2 Detection of hormones

The contents of various hormones such as auxin, cytokinins (CK), abscisic acid (ABA), jasmonates (JA), salicylic acid (SA), gibberellins (GA), ethylene (ETH), strigolactones (SL), and melatonin (MLT) were detected using MetWare platform based on the AB Sciex QTRAP 6500 LC-MS/MS platform (UPLC, ExionLC™ AD, and MS, QTRAP^®^ 6500, https://sciex.com.cn/). The detail detection and analysis procedure refers to the published method (Zhang et al., 2020).

### 2.3 RNA extraction and Illumina sequencing

The extraction and purification of total RNA, cDNA libraries, and high-throughput sequencing were performed and analyzed by Novogene Bioinformation Technology Co., Ltd., Beijing, China. The reference genome was the *Citrus sinensis* genome (https://www.citrusgenomedb.org/organism/Citrus/sinensis) and the RNA-seq data was mapped using HISAT2 software (Kim et al., 2015). The detail detection and analysis procedure refers to the published method (Wang et al., 2021). Hormones and the DEGs in the transcriptome data were chosen for integrative analysis. Pearson correlation coefficients and *p*-values for hormones and DEGs data integration were calculated using the Spearman method.

### 2.4 Quantitative real-time PCR (qRT-PCR) validation

The total RNA of citrus pericarp was extracted by the Quick RNA isolation kit (Huayueyang, Beijing, China). The cDNA was synthesized using a Hifair^®^ III 1st strand cDNA synthesis kit (Yeasen, Shanghai, China). Ten DEGs were selected for verification with specific primers by LightCycle 96 Real-Time PCR System (Roche, Switzerland) (**Supplementary Table S11**). Hieff^®^ qPCR SYBR Green Master Mix (Yeasen, Shanghai, China) was used for qRT-PCR. The amplification system and program refer to the product description. The expression level was analyzed by the 2^−ΔΔct^ method with a reference gene, *β-actin*.

### 2.5 Statistical analysis

SPSS 22.0 was utilized for statistical analysis, and the levels of significance were determined using the least significant difference. (*p*-value < 0.05).

## 3 Results

### 3.1 Phenotypic analysis of fruit appearance

**Figure 1:**
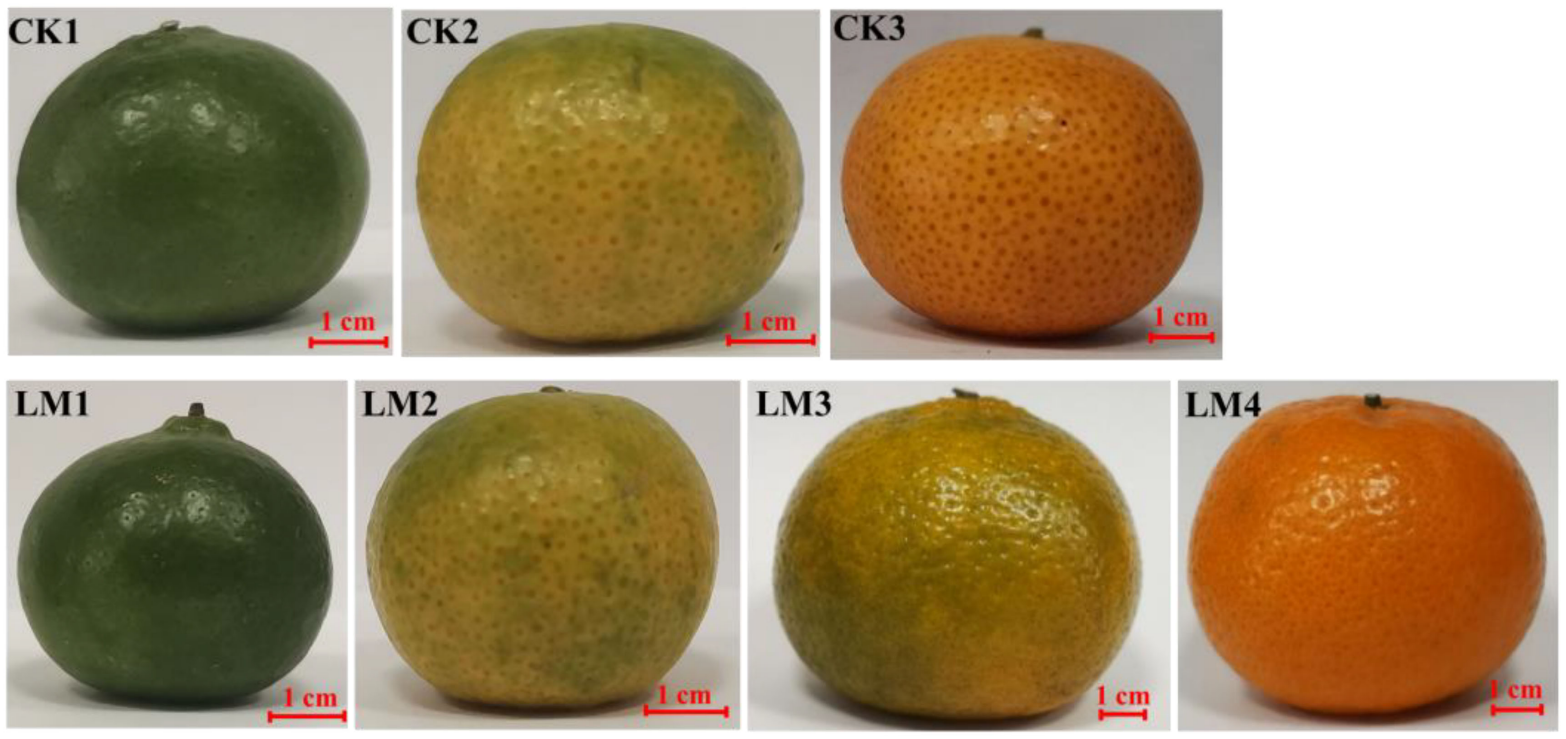
Fruit phenotypes at the different development stages of two citrus varieties, Mandarin ‘Chunhongtangju’ (LM) and ‘Shatangju’ (CK).

**Figure 2:**
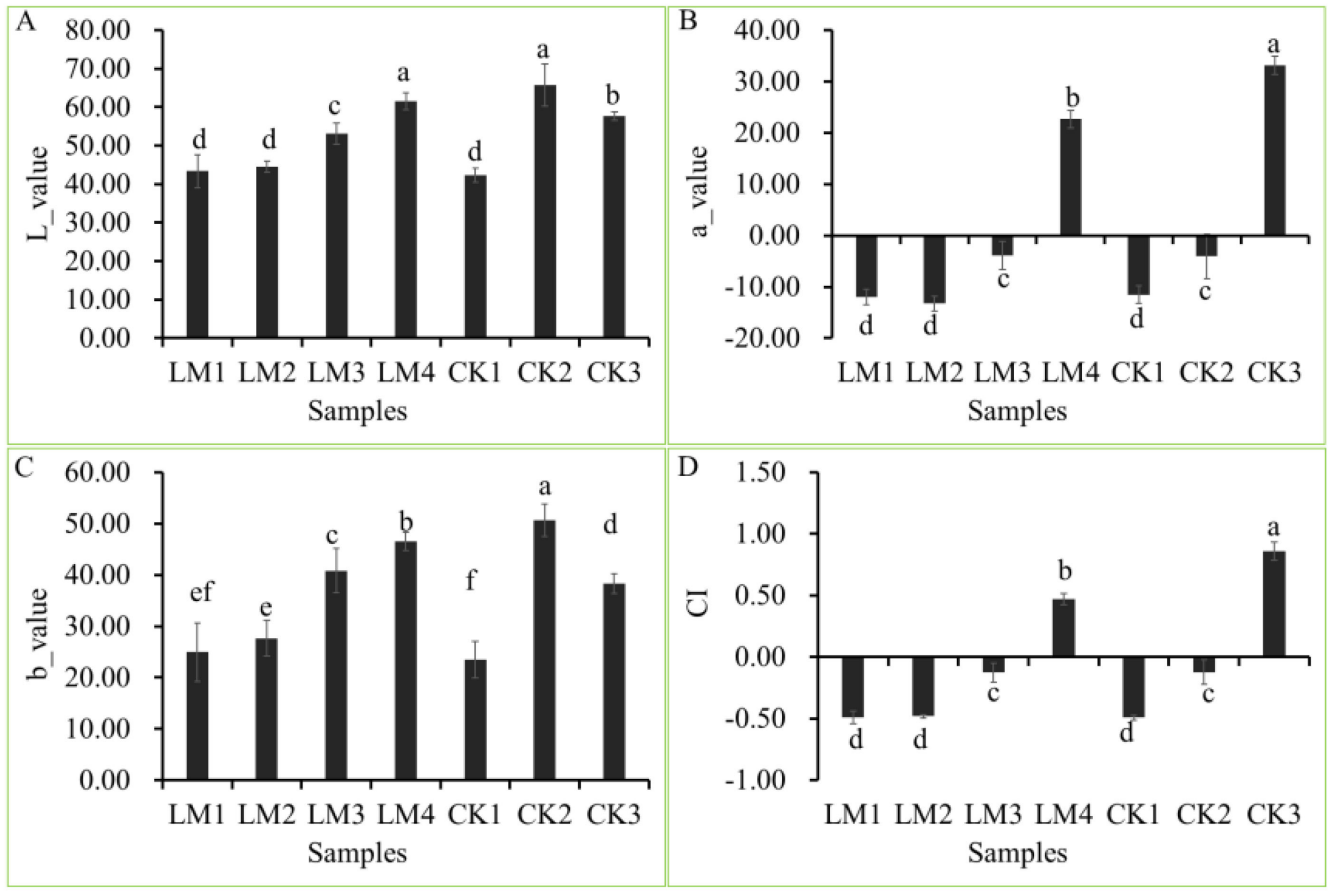
Difference between the four-color parameters at different development stages of Mandarin ‘Chunhongtangju’ (LM) and ‘Shatangju’ (CK). **(A)** L-value of colar patameters. **(B)** a-value of colar patameters. **(C)** b-value of colar patameters. **(D)** CI is the color index. Values indicate the *p*-value of the Student test: *p*-value < 0.05. Different lowcase letters above columns indicate statistical differences at P < 0.05.

The fruits used in the present study were harvested at different development stages of two citrus varieties, Mandarin ‘Shatangju’, and ‘Chunhongtangju’ and the coloration was detected by a colorimeter (**Figures 1**, **2**). For the four chromaticity parameters, the L-index indicates the darkness and brightness of the fruit pericarp, and the larger the L-index, the brighter the sample surface. The a-index indicates the red-green difference, with positive values indicating red and negative values indicating green. The b-index represents the yellow-blue difference, and the larger the b-index, the darker the yellow color of the fruit. CI represents the color saturation of the fruit color; the larger the CI value, the brighter the color. In the first developmental stage of the two citrus varieties, CK1 and LM1, all parameters (L, a b, and CI) were consistent without significant differences (**Figure 2**). Interestingly, the four chromaticity parameters remain at the level in the second stage of Mandarin ‘Chunhongtangju’, but the values of the four chromaticity parameters have far exceeded those of the first stage in Mandarin ‘Shatangju’ (**Figure 2**). At the third development stage, the chromaticity parameters of Mandarin ‘Chunhongtangju’ were still lower than those of Mandarin ‘Shatangju’ (**Figure 2**). In the third stage, the fruits of Mandarin ‘Shatangju’ were ripe for harvest. When the fruits of Mandarin ‘Chunhongtangju’ were ripe at the LM4 stage, the L- and b-index exceeded the index level of CK3 but the a-index and CI were lower (**Figures 1**, **2**).

### 3.2 Quantitative analysis of hormones in all samples

**Figure 3:**
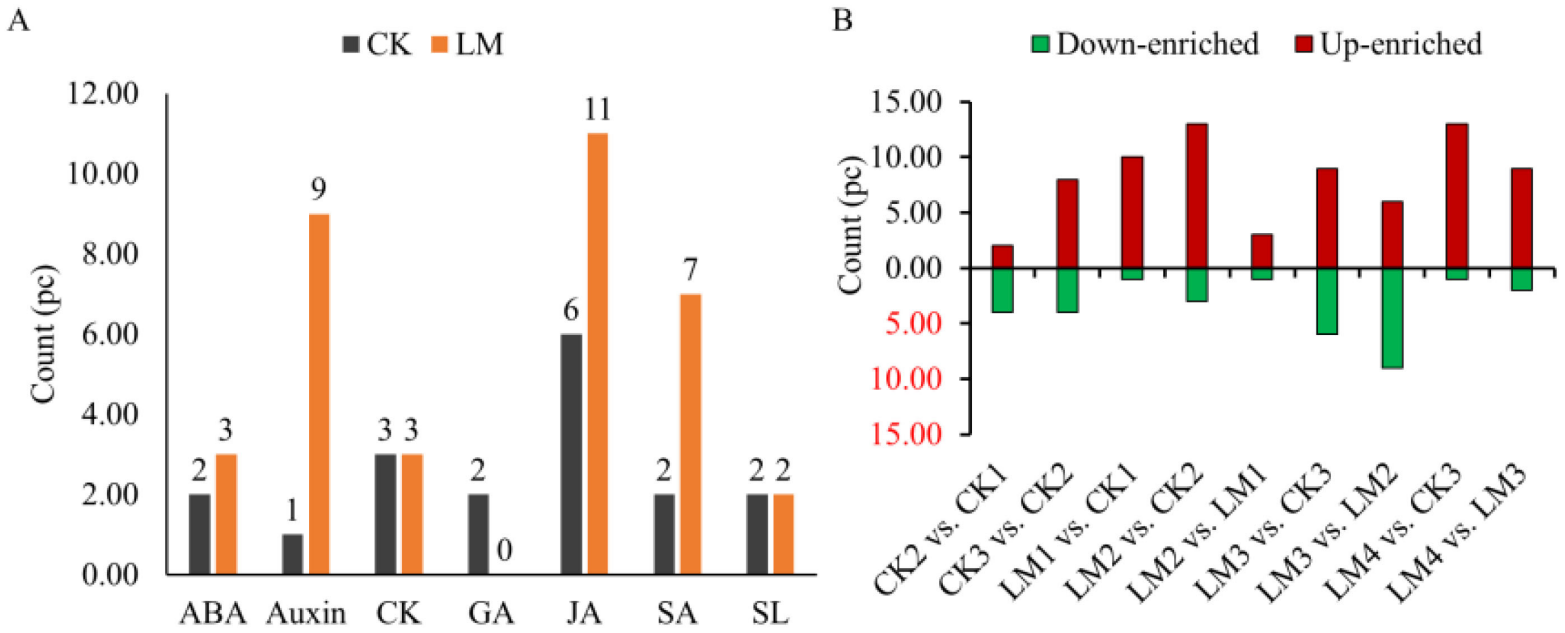
Quantitative statistics of hormones detected in all pericarp samples and comparison groups. **(A)** The total enriched count of each class. **(B)** down_enriched, and up_enriched count of hormones in different comparison groups.

**Figure 4:**
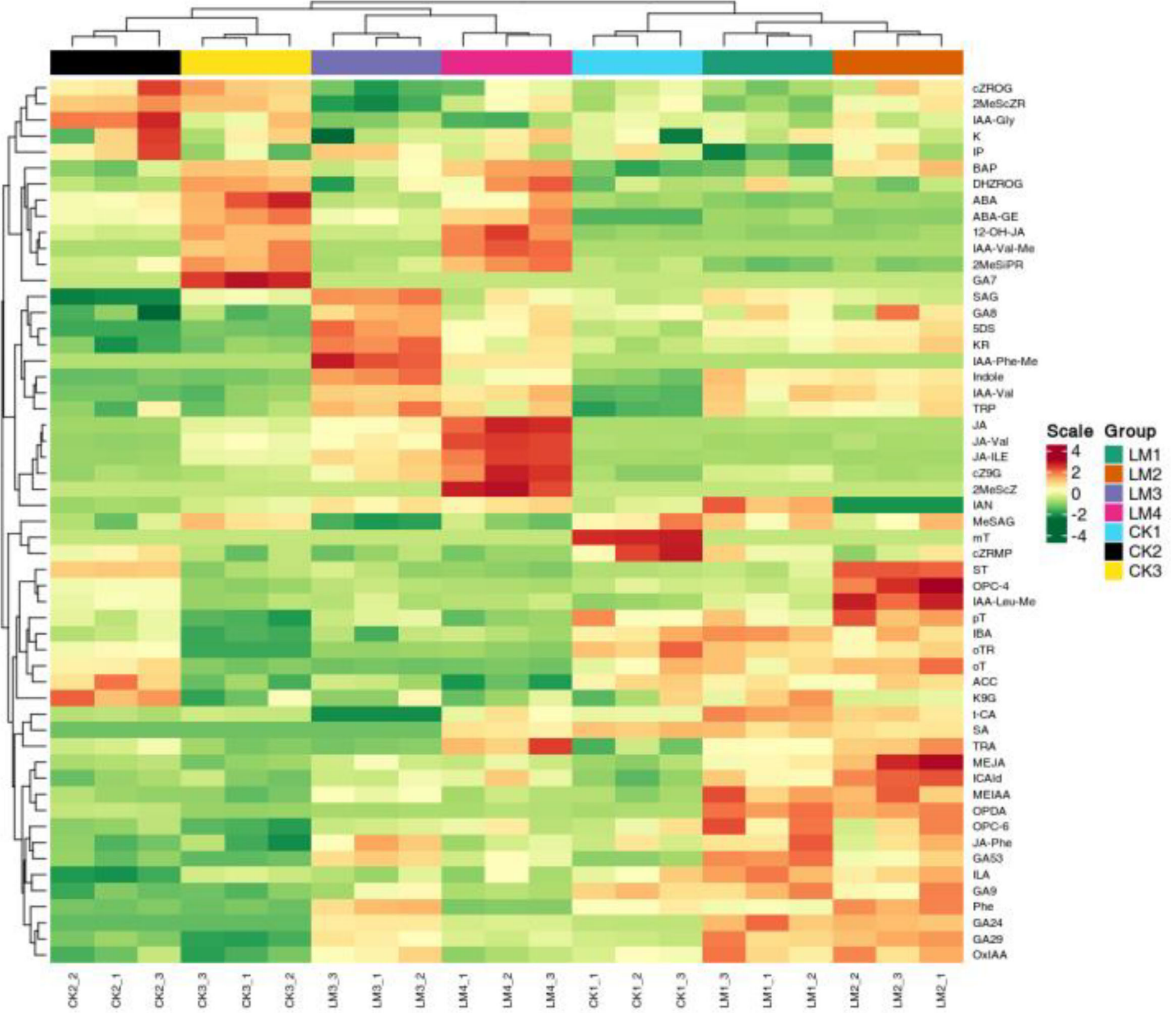
The HCA of hormones detected in all pericarp samples.

Nine classes of hormones including auxin, cytokinins (CK), abscisic acid (ABA), jasmonates (JA), salicylic acid (SA), gibberellins (GA), ethylene (ETH), strigolactones (SL), and melatonin (MLT) were identified and measured by UPLC. A total of fifty-five hormones belonging to eight classes were identified in the CK and LM groups (**Supplementary Table S1**). The results indicated that auxin (L-tryptophan, TRP; indole), SA (L-phenylalanine, Phe), and ABA (abscisic acid, ABA; ABA-glucosyl ester, ABA-GE) were the most abundant hormones in two cultivars samples (**Supplementary Table S1**). In the Mandarin ‘Shatangju’ cultivars, several hormones, including ABA, Auxin, CK, GA, JA, SA, and SL, were found to be actively involved in the development and ripening process of fruits from CK1 to CK3 (**Figure 3A**). In Mandarin ‘Chunhongtangju’, the major hormones were similar to the Mandarin ‘Shatangju’, except for GA which did not show a significant difference from LM1 to LM4 (**Figures 3A**, **4**; **Supplementary Table S2**). During the development and ripening process, there were significant differences in most classes of hormones. The top two up-enriched hormones in Mandarin ‘Chunhongtangju’ pericarps included gibberellin A24 (Log2FC =1.73), cis (+)-12-oxophytodienoic acid (Log2FC =2.41) in LM1 *vs.* CK1; L-phenylalanine (Log2FC =1.35) and cis (+)-12-oxophytodienoic acid (Log2FC =1.79) in LM2 *vs.* CK2; Gibberellin A53 (Log2FC =1.76) and 5-Deoxystrigol (Log2FC =2.94) in LM3 *vs.* CK3); Jasmonic acid (Log2FC =1.77) and 5-Deoxystrigol (Log2FC =2.35) in LM4 *vs.* CK3 (**Figures 3B**, **4**; **Supplementary Table S2**). The content of N-(3-Indolylacetyl)-L-valine (auxin) in Mandarin ‘Chunhongtangju’ from LM1 to LM4 was consistently higher than those in Mandarin ‘Shatangju’ from CK1 to CK3 (**Supplementary Table S2**). The deficiency of hormones in Mandarin ‘Chunhongtangju’ fruits was mainly manifested in meta-Topolin (CK) in the LM1 stage; 3-Indoleacetonitrile (auxin) in LM2 stage; Indole-3-acetyl-L-valine methyl ester (auxin), Gibberellin A7 (GA), and trans-Cinnamic acid (SA) in LM3 stage; Gibberellin A7 (GA) in LM4 stage. These hormones were not detected in the late-maturing pericarps.

### 3.3 Differential expression of genes in the three comparisons

**Figure 5:**
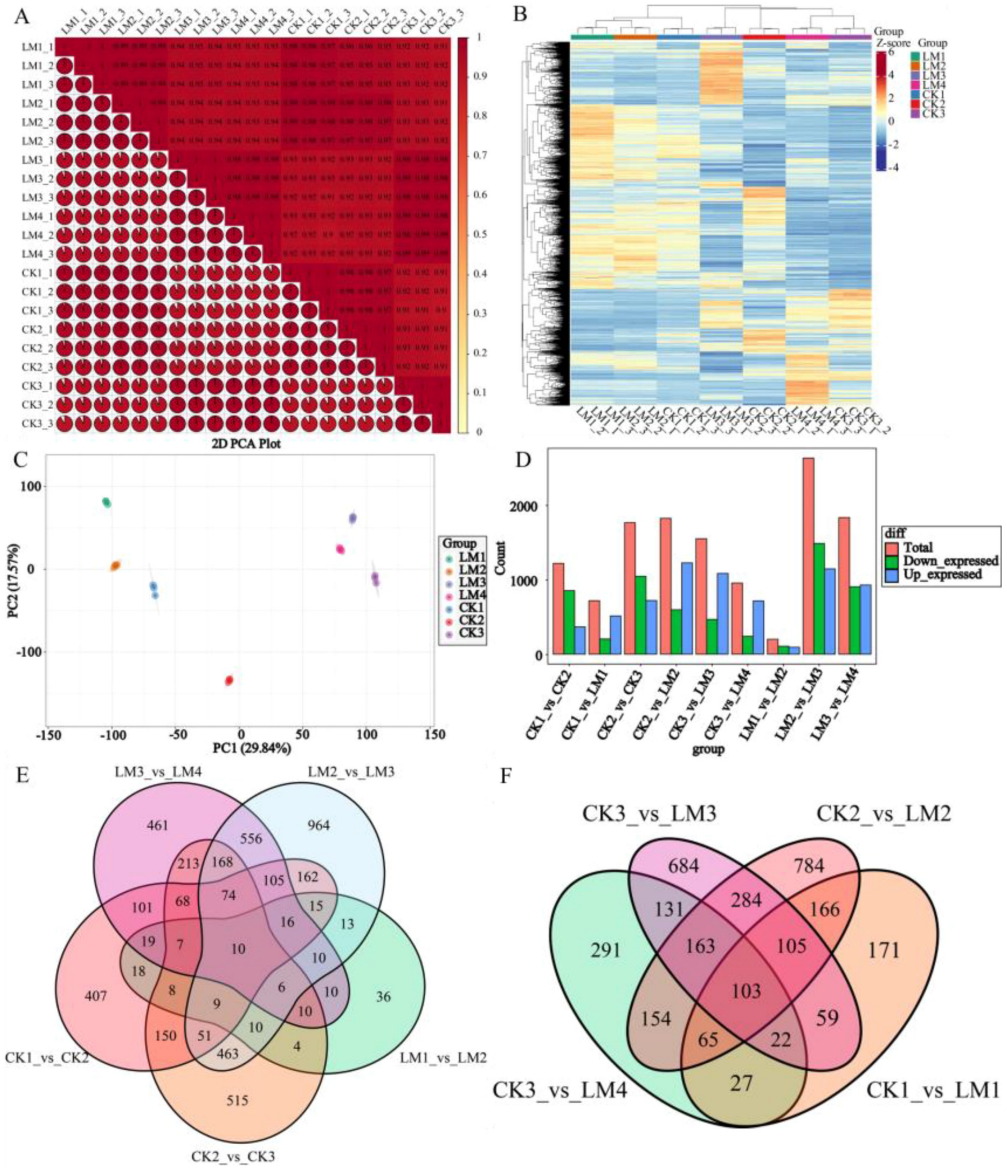
The overall gene expression of all samples and statistics of DEGs in different comparison groups. **(A)** Correlation heatmap of all samples (R^2^>0.8). **(B)** The hierarchical cluster analysis (HCA). **(C)** Principal component analysis (PCA) score plots. **(D)** Statistics of down/up-regulated genes. **(E)** Venn diagrams of DEGs in the five comparison groups at different development stages. **(F)** Venn diagrams of DEGs in the four comparison groups at the same development stages.

To compare the differential gene expression between the pericarps of two different varieties at various development stages, transcriptomic analyses were conducted across seven stages of fruit development. RNA-Seq produced 73.75-99.14 (97.16-97.84%), 84.27-95.77(97.33-97.62%), 57.77-91.88(96.47-97.51%), and 73.67-78.19(98.02-98.29%), 68.25-82.43(97.99-98.16%), 59.86-88.70 (97.32-97.61%), 71.37-99.54(96.29-97.73%) million clean reads from CK1 to CK3 and LM1 to LM4 samples cDNA libraries after stringent quality checks and data clean-up, respectively (**Supplementary Table S3**). In total, 67.99-91.76, 77.28-87.83, 53.26-84.73, and 67.87-72.02, 62.86-75.91, 55.31-81.86, 65.63-92.16 million reads were mapped to the *Citrus sinensis* genomic database (http://citrus.hzau.edu.cn), with match ratios in the ranges of 92.15-92.56%, 91.66-91.72%, 92.21-92.51%, and 92.11-92.38%, 92.06-92.11%, 92.30-92.43%, 91.96-92.59% in CK1 to CK3 and LM1 to LM4 samples, respectively (**Supplementary Table S3**). A total of 21,926, 22,041, 21,487, and 2,1854, 21,843, 21,823, and 21,639 genes were identified and detected in CK1/2/3 and LM1/2/3/4 samples, respectively (**Supplementary Table S4**). A high correlation coefficient (R^2^>0.99) of gene expression between biological replicates indicated the effectiveness of the data (**Figure 5A**). The principal component analysis (PCA) and HCA show the large separation of trends among different treatment groups and little intragroup variation (**Figures 5B, C**). Therefore, these results suggested that all transcriptomic data in the present study demonstrate good repeatability and reliability.

With the filter criteria of |log_2_FoldChange| ≥1 and false discovery rate (FDR) < 0.05, In total, 5,221 differentially expressed genes (DEGs) were identified through three stages in Mandarin ‘Shatangju’ and four stages in Mandarin ‘Chunhongtangju’ (**Supplementary Table S5**). And there were 1220, 718, 1766, 1824, 1551, 956, 201, 2632, and 1834 differentially expressed genes (DEGs) detected in the nine comparisons, CK1 *vs.* CK2, CK1 *vs.* LM1, CK2 *vs.* CK3, CK2 *vs.* LM2, CK3 *vs.* LM3, CK3 *vs.* LM4, LM1 *vs.* LM2, LM2 *vs.* LM3, and LM3 *vs.* LM4, respectively, of which 854, 205, 1046, 597, 467, 241, 107, 1486, and 905 DEGs were up-regulated, and 366, 513, 720, 1227, 1084, 715, 94, 1146, and 929 DEGs were down-regulated in the nine comparison groups, respectively (**Supplementary Table S5**; **Figure 5D**). Venn diagram analysis shows that 10 DEGs were common to the five comparison groups during different development stages (**Figure 5E**). During the fruit development and ripening, some differentially expressed genes coexisted in the two varieties, including 18 DEGs for stage 1 to stage, 2, 463 DEGs for stage 2 to stage 3, and 213 DEGs between stage 2 to stage 3 of Mandarin ‘Shatangju’ and 3 to 4 of Mandarin ‘Chunhongtangju’ (**Figure 5E**). They have their DEGs when the fruits develop to the same stage. No matter what stage of development, 103 DEGs coexisted in the two varieties (**Figure 5F**). The 103 DEGs are mainly involved in oxidative phosphorylation, steroid biosynthesis, ABC transporters, phenylpropanoid biosynthesis, protein processing in the endoplasmic reticulum, amino sugar and nucleotide sugar metabolism and MAPK signaling pathway–plant.

**Figure 6:**
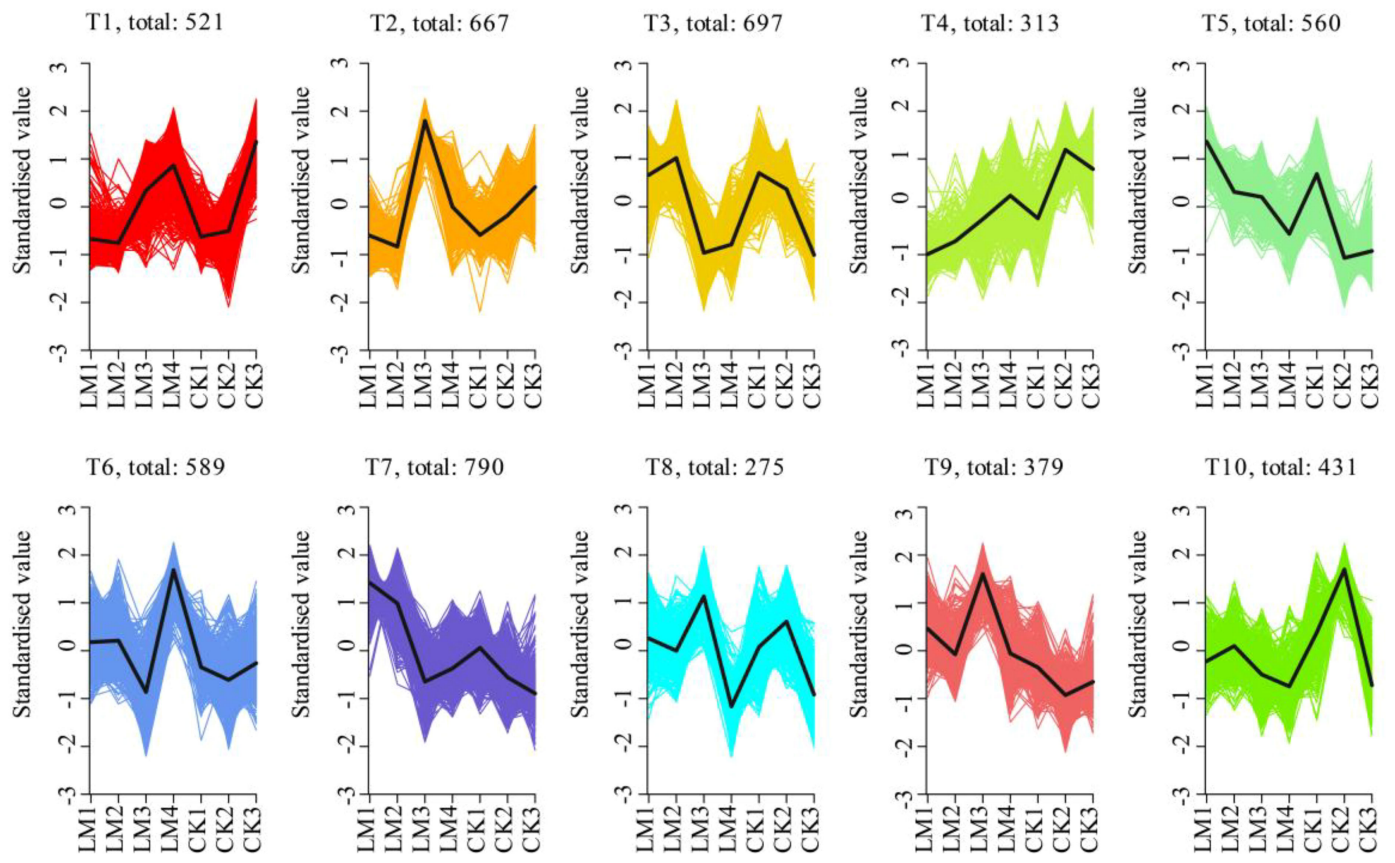
K-means clustering analysis of all DEGs in all samples.

In total, 5221 differentially expressed genes (DEGs) were identified during the four development stages (**Supplementary Table S6**). Further, k-means clustering analysis exhibited 10 distinct clusters (T1–T10) corresponding to four different developmental stages of two citrus cultivars: CK_2 (T4 and T10), CK3 (T1), LM1 (T5 and T7), LM2 (T3), LM3 (T2, T8-T9), and LM4 (T6) (**Figure 6**; **Supplementary Table S6**). It suggests that the high-expression patterns of identified genes were diverse throughout fruit development and ripening of two citrus cultivars (**Figure 6**; **Supplementary Table S6**). The CK1 did not show high-expression genes. While inspecting the potential roles of DEGs, the high-expression genes in CK2 (shown by T4 and T10), and LM2 (shown by T3) were mainly involved in ABC transporters, MAPK signaling pathway, plant hormone signal transduction, and plant-pathogen interaction and also some high-expression genes in LM2 were involved in glutathione, starch and sucrose metabolism, and ubiquitin-mediated proteolysis; high-expression genes in LM1 (shown by T5 and T7) were mainly involved in plant hormone signal transduction, ABC transporters MAPK signaling pathway, photosynthesis, carotenoid, and phenylpropanoid biosynthesis; the highest expression genes from CK/LM2 to CK/LM3 were mainly involved in phenylpropanoid biosynthesis, plant hormone signal transduction, and plant-pathogen interaction but some high-expression genes in LM3 also were involved in some biosynthesis and metabolism processes that were similar to the LM2, such as MAPK signaling pathway, and starch and sucrose metabolism. These mean that the fruits at the LM3 stage were developing like LM2. When the fruits of Mandarin ‘Chunhongtangju’ were developed from LM3 to LM4, the high-expression genes were mainly involved in plant-pathogen interaction, plant hormone signal transduction, MAPK signaling pathway, and ABC transporters that were similar to the Mandarin ‘Shatangju’ fruits from CK2 to CK3 (**Figure 6**; **Supplementary Table S6**). This is in solid agreement with the role of hormones in the fruit development of Mandarin ‘Chuntongtangju’.

The Gene Ontology (GO) analysis was conducted to investigate the roles of genes related to fruit development and maturation in the biological process (BP), cell component (CC), and molecular function (MF) terms. For the changes in gene expression levels during fruit development and ripening, the GO analysis of 1220 DEGs in CK1 *vs.* CK2 comparison was mainly enriched in biological process and cellular component including photosynthesis-related process, generation of precursor metabolites and energy, pigment metabolic process, chloroplast thylakoid membrane, plastid thylakoid membrane, and photosynthetic membrane (**Supplementary Figure S1A**). 1766 DEGs in CK2 *vs.* CK3 comparison were mainly enriched in secondary metabolic processes (belong to BP) such as phenylpropanoid biosynthetic process, flavonoid biosynthetic process, and suberin biosynthetic process, and some apoplast-related cellular components, and molecular functions such as glucosyltransferase activity, cytoskeletal motor activity, tetrapyrrole binding, and heme binding were also actively (**Supplementary Figure S1B**). 201 DEGs in LM1 *vs.* LM2 were mainly enriched in molecular functions such as carboxylic ester hydrolase activity, galactolipase activity, and phospholipase activity (**Supplementary Figure S1C**). 2632 DEGs in the LM2 *vs.* LM3 were mainly enriched in the biological process. The response processes to ethylene, hypoxia, phosphorelay, molecular transducer, and transmembrane signaling receptor activity were activated (**Supplementary Figure S1D**). 1834 DEGs detected in the LM3 vs. LM4 were mainly enriched in biological processes and molecular functions such as the xyloglucan-glucan metabolic process, plant-type cell wall organization biogenesis, glucosyltransferase, xyloglucan-xyloglucosyl transferase, and hexosyltransferase activity (**Supplementary Figure S1E**). When the fruits reach the first development stage of development, the differences in transcription levels between the two varieties are mainly manifested in secondary metabolism (e.g. phenylpropanoid biosynthetic process, phenol/benzene-containing compound metabolic process, and aminoglycan metabolic process) and hormone metabolism (e.g. salicylic acid metabolic process); in the second and third development stages, the differences in transcription levels between the two varieties are mainly manifested in photosynthesis and energy synthesis (e.g. photosynthesis, chloroplast thylakoid membrane, plastid thylakoid membrane); as the late-ripening fruits reach the fourth development stage, the differences in transcriptional levels compared to Mandarin ‘Shatangju’ fruits at the third stage were mainly in terms of cellular-related biological process (e.g. cellular response to hypoxia/oxygen levels) (**Supplementary Figure S2**).

KEGG analysis (*p*-value < 0.05) revealed that all DEGs of two CK comparison groups were mainly enriched in 12 metabolic processes that involved multiple aspects, such as energy metabolism, cofactors and vitamins metabolism, carbohydrate metabolism, secondary metabolites metabolism, and lipid metabolism; all DEGs of three LM comparisons groups were mainly enriched in 8 metabolic processes that involved in energy metabolism, signal transduction, and other secondary metabolites biosynthesis (**Supplementary Figure S3**). The top three KEGG pathways (adjust *p*-value <0.05) were photosynthesis, biosynthesis of secondary metabolites, and photosynthesis-antenna proteins in CK1 *vs.* CK2; phenylpropanoid biosynthesis, plant-pathogen interaction, biosynthesis of secondary metabolites in CK2 *vs.* CK3; photosynthesis-antenna proteins in LM1 *vs.* LM2; plant-pathogen interaction, MAPK signaling pathway-plant, and plant hormone signal transduction in LM2 *vs.* LM3; plant hormone signal transduction, plant-pathogen interaction, and MAPK signaling pathway-plant LM3 *vs.* LM4 (**Supplementary Table S7**). During the development of fruits, the differences in transcription levels between the two varieties are mainly manifested in signal transduction, lipid metabolism, biosynthesis of other secondary metabolites, energy metabolism, environmental adaptation, and other aspects. These aspects were specifically manifested in cutin, suberin, wax, and phenylpropanoid biosynthesis, photosynthesis, plant-pathogen interaction, carbon fixation, and plant hormone signal transduction. Among these up-regulated DEGs of CK1 *vs.* LM1, CK2 *vs.* LM2, CK3 *vs.* LM3, and CK3 *vs.* LM4, 35 protein kinase domain genes, 27 protein tyrosine and serine/threonine kinase genes, 24 cytochrome P450 genes, 14 chlorophyll A-B binding protein genes, 14 peroxidase gene, 13 leucine-rich repeat genes, 13 transferase family genes, 13 WRKY DNA -binding domain genes, 10 UDP-glucoronosyl and -glucosyl transferase genes were found; and the down-regulated DEGs mainly included 12 protein kinase domain genes, 6 auxin-responsive protein genes, 6 leucine-rich repeat gene, 6 transferase family genes, 5 OG-Fe(II) oxygenase superfamily genes (**Supplementary Table S7**; **Supplementary Figure S4**). The qRT-PCR results of ten candidate genes showed that five genes (Cs_ont_5g030100, Cs_ont_2g022940, Cs_ont_9g010690, Cs_ont_2g022890, and novel.698) were down-regulated in late-maturing varieties, while four genes (Cs_ont_7g002060, Cs_ont_1g017990, Cs_ont_3g029910, and Cs_ont_2g033670) were up-regulated. The overall validation results are consistent with the RNA-Seq results.

### 3.4 Differentially expressed transcription factors in the transcriptome

**Figure 7:**
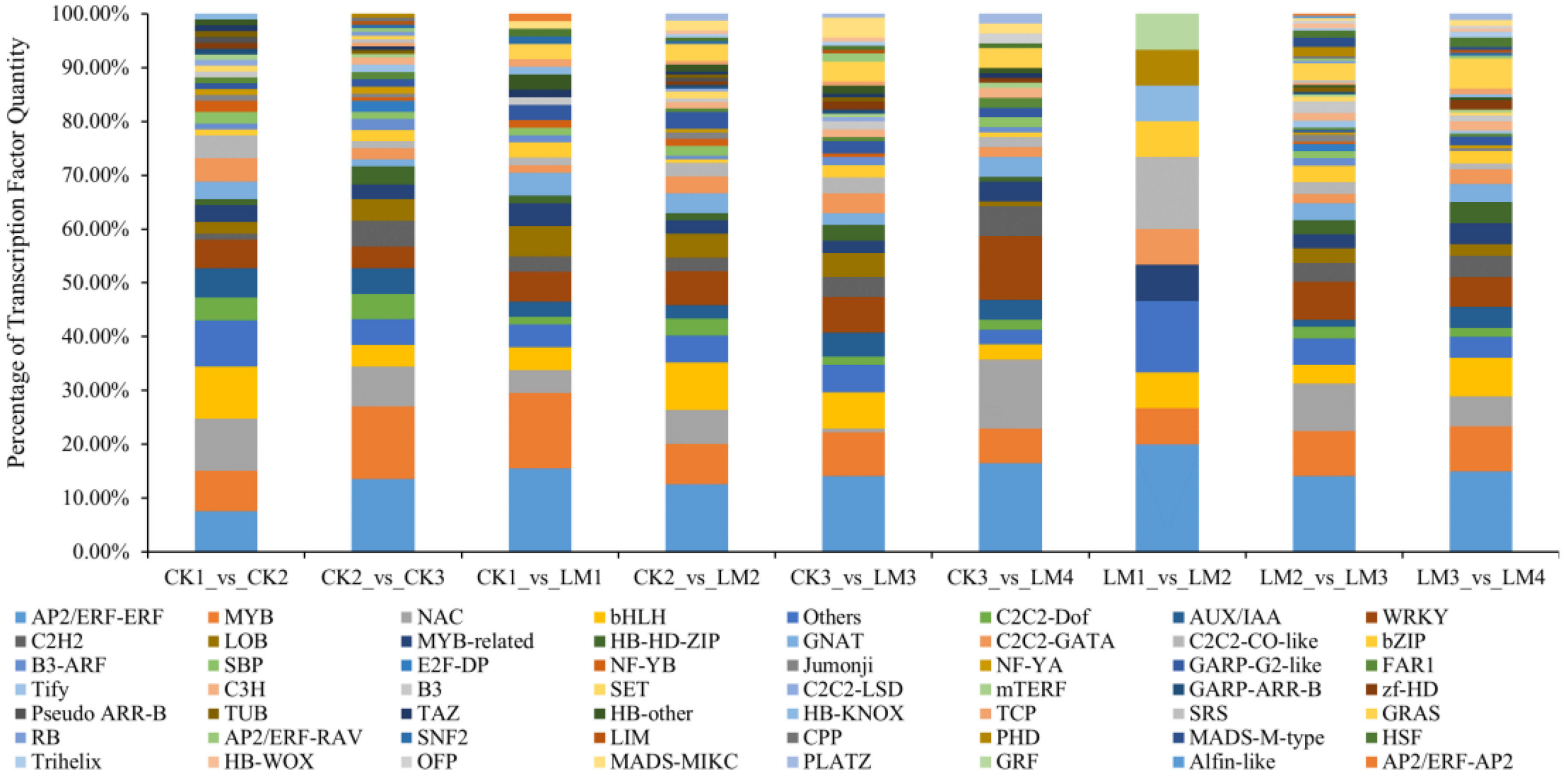
Differentially expressed transcription factors (DETFs) in the comparison groups.

In total, 93 and 148 differentially expressed transcription factors (DETFs) that belong to 46 TF families such as AP2/ERF-ERF, MYB, NAC, bHLH, C2C2-Dof, and others were identified in CK1 *vs*. CK2, and CK2 *vs*. CK3 and 15, 228, 183 DETFs in LM1 *vs*. LM2, LM2 *vs*. LM3, and LM3 *vs*. LM4 mainly belonged to AP2/ERF-ERF, MYB, NAC, bHLH, WRKY, and others TF families (**Figure 7**; **Supplementary Table S8**). During the four development stages of fruits, 71 DETFs in CK1 *vs*. LM1, 160 DETFs in CK2 *vs*. LM2, 137 DETFs in CK3 *vs*. LM3, and 109 DETFs in CK3 *vs*. LM4 displayed differential transcriptional regulation characteristics between two citrus varieties. The AP2/ERF-ERF (e. g. Cs_ont_1g012050, Cs_ont_5g024690, and Cs_ont_4g022340) and AUX/IAA (e. g. Cs_ont_3g019530, Cs_ont_4g002310, and Cs_ont_4g004790) family were the mainly DETFs comparing between two citrus varieties. The down-regulated TF of Mandarin ‘Chunhongtangju’ included 5 AP2/ERF-ERF (Cs_ont_8g024590, Cs_ont_5g026350, Cs_ont_5g024690, Cs_ont_1g003740, and Cs_ont_1g012050), 2 AUX/IAA (Cs_ont_4g004790, and Cs_ont_4g002310), 2 MYB (Cs_ont_8g025330, and Cs_ont_9g027950), and so on. These TF may have affected the development of the ‘Chunhongtangju’ fruits and need to be further researched.

### 3.5 Integrative analysis of transcriptome and hormone

**Figure 8:**
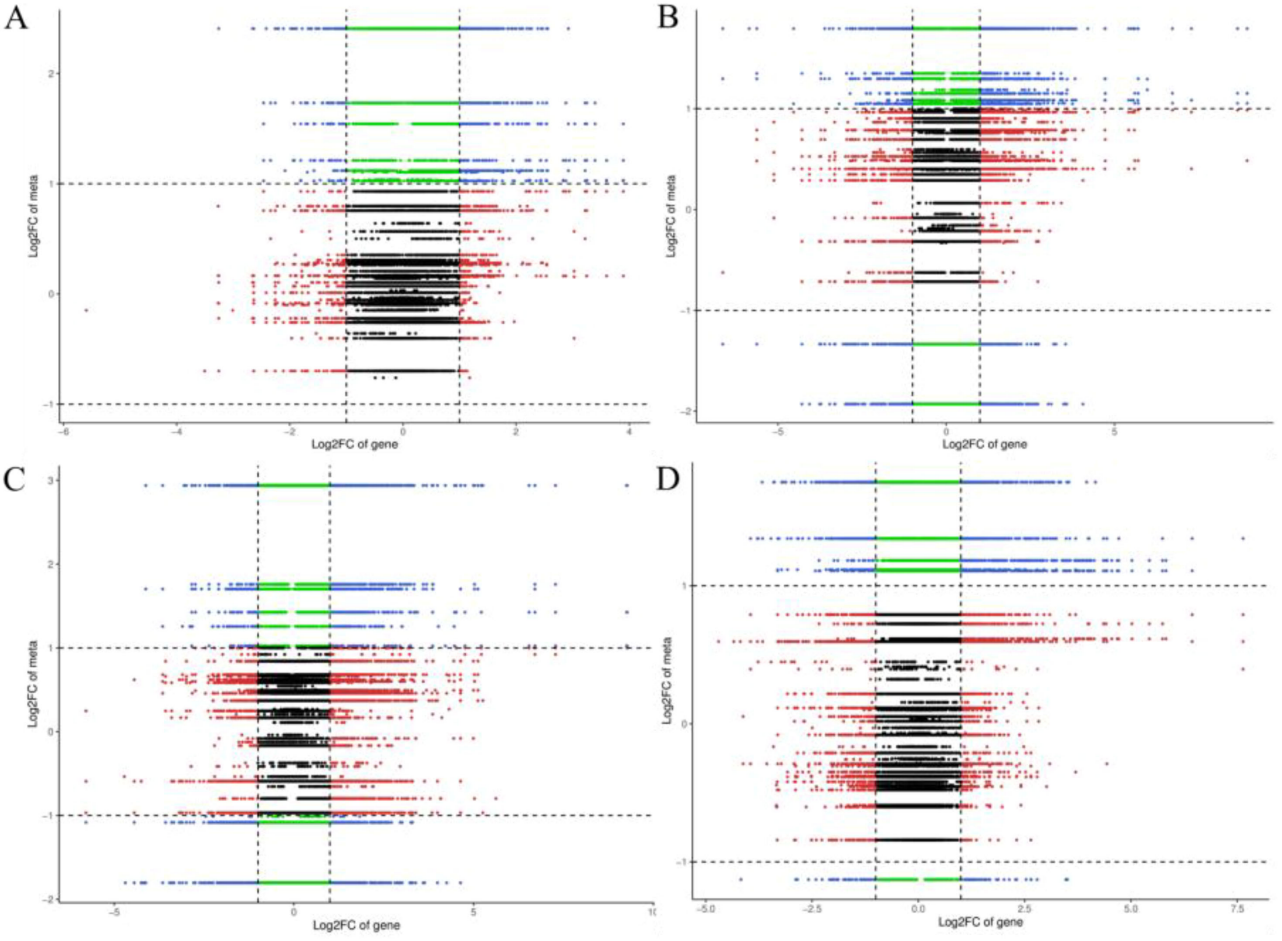
Nine quadrants of DEGs and hormones in the comparison groups. **(A)** Nine quadrants of CK1 *vs.* LM1. **(B)** Nine quadrants of CK2 *vs.* LM2. **(C)** Nine quadrants of CK3 *vs.* LM3. **(D)** Nine quadrants of CK3 *vs.* LM4.

**Figure 9:**
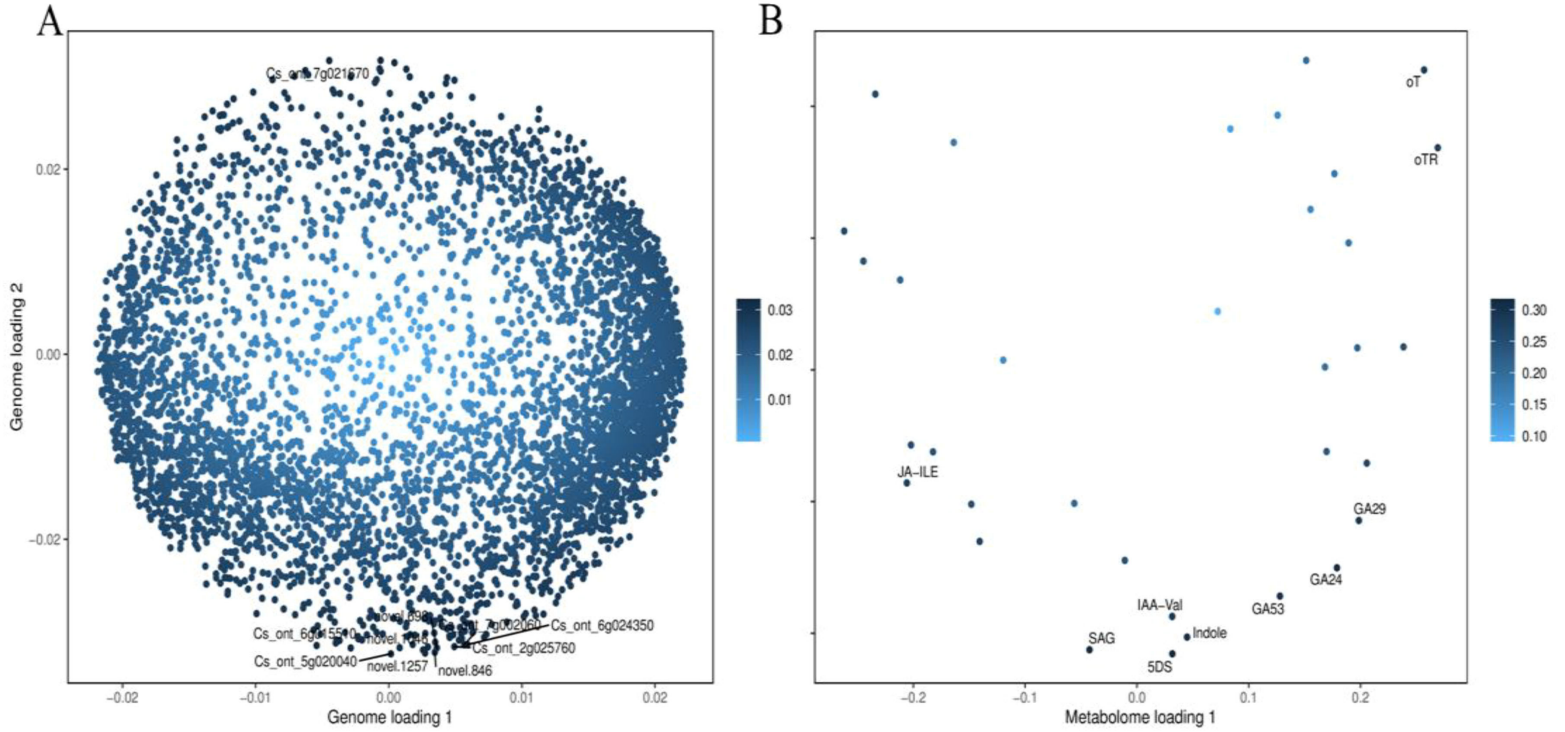
OPLS of DEGs and hormones in the comparison groups. **(A)** OPLS of DEGs. **(B)** OPLS of the hormone.

The integrative analysis of transcriptome and hormone data investigates the association between DEGs and hormones of fruit pericarps at the same development stage. Results showed that there were 548,1547, 1156, and 713 genes highly positive or negative corrected to 10, 15, 12, and 11 kinds of hormones in CK1 *vs*. LM1, CK2 *vs*. LM2, CK3 *vs*. LM3, and CK3 *vs*. LM4, respectively (coefficient ≥0.8, *p*-value≤ 0.05, **Figure 8**; **Supplementary Table S9**). The highly corrected hormones included ABA-GE (ABA-glucosyl ester), IAA-Val (n-(3-indolyl acetyl)-l-valine), TRP (l-tryptophan), TRA (tryptamine), MEIAA (methyl indole-3-acetate), Indole (indole), GA24 (gibberellin A24), GA53 (gibberellin A53), OPDA (cis(+)-12-oxophytodienoic acid), ABA (abscisic acid), GA29 (gibberellin A29), MEJA (methyl jasmonate), OPC-4 (3-oxo-2-(2-(Z)-pentenyl) cyclopentane-1-butyric acid), t-CA (trans-cinnamic acid), SA (salicylic acid), Phe (L-phenylalanine), 5DS (5-deoxystrigol), oTR (ortho-tooling riboside), 12-OH-JA (12-hydroxy jasmonic acid), MeSAG (2-methoxycarbonyl phenyl beta-d-glucopyranoside), cZ9G (cis-zeatin-9-glucoside), JA-ILE (jasmonate-l-isoleucine), JA (jasmonic acid), JA-Val (n-[(-)-jasmonoyl]-(L)-valine) in the four comparison groups. There was a total of 629 DEGs in CK1 *vs*. LM1, CK2 *vs*. LM2, CK3 *vs*. LM3, and CK3 *vs*. LM4 corrected to the above hormones, and these genes mainly involved in carbohydrate, energy, lipid, cofactors, and vitamins metabolism, biosynthesis of other secondary metabolites (e.g. phenylpropanoid biosynthesis), and signal transduction (e.g. MAPK signaling pathway-plant, plant hormone signal transduction) such as AP2/ERF domain-containing protein (Cs_ont_9g008010 and Cs_ont_5g011900) and auxin response factor (Cs_ont_4g004790, Cs_ont_4g002310, and Cs_ont_5g011250 in **Supplementary Table S10**, **Supplementary Figure S5**. OPLS statistical method was used to screen the top ten genes or hormones that had a greater influence on the two omics data. The top gene included novel.1257, novel.846, Cs_ont_5g020040, novel.1046, Cs_ont_7g021670, Cs_ont_2g025760, Cs_ont_6g015510, novel.698, Cs_ont_6g024350, and Cs_ont_7g002060; the top hormones were 5-deoxystrigol (5DS), salicylic acid 2-o-β-glucoside (SAG), gibberellin A24 (GA24), indole (Indole), gibberellin A53 (GA53), gibberellin A29 (GA29), n-(3-indolylacetyl)-l-valine (IAA-Val), ortho-topolin (oT), ortho-topolin riboside (oTR), and jasmonoyl-l-isoleucine (JA-ILE) in **Figure 9**.

## 4 Discussion

### 4.1 Phenotypic differences in fruit development of two citrus varieties

The color transformation of citrus pericarps mainly includes four stages, degreening, yellowing, orange-turning, or red-turning, in which chlorophyll degradation and carotenoid synthesis are mainly involved (Rodrigo et al., 2013; Lu et al., 2017). In the present study, the fruits were harvested at different development stages of two citrus varieties. In the first and second developmental stages of the late variety Mandarin ‘Chunhongtangju’, all chromaticity parameters (L, a b, and CI) were similar without significant differences. The fruits remained green. In the third stage, the fruits start to go through color-break and the fruits do not ripen until the fourth stage in the late-maturing Mandarin ‘Chunhongtangju’. When the fruits develop toward maturity, citrus chlorophyllase dynamics can be affected by ethylene, and the citrus fruit starts to color-break (Shemer et al., 2008). The fruits of Mandarin ‘Shatangju’ start to color-break in the second stage and the fruits ripen in the third stage but ‘Chunhongtangju’ fruits have an extra period to ripen. Therefore, the mature period of the late-maturing Mandarin ‘Chunhongtangju’ is significantly delayed than ‘Shatangju’.

### 4.2 Hormone profiles of the citrus pericarp at different developmental stages

Plant hormones play a crucial role in the growth and development of plants, including the formation of various tissues such as roots (Xie et al., 2021), flowers (Chandler, 2011), and leaves (Liu et al., 2022). For example, GA can induce cell elongation (Gapper et al., 2013), and ethylene contributes to the development of non-climacteric fruits (Osorio et al., 2013; Kumar et al., 2014). In this study, ABA, Auxin, CK, JA, SA, and SL were actively carried out for the development of fruits in Mandarin ‘Shatangju’ and ‘Chunhongtangju’ but not including GA in Mandarin ‘Chunhongtangju’. During the ripening process, the content of most hormones in Mandarin ‘Chunhongtangju’ pericarps at the same period was higher than that in Mandarin ‘Shatangju’ pericarps such as gibberellin A24, cis (+)-12-oxophytodienoic acid, L-phenylalanine and indole. The main hormones with a lower content in Mandarin ‘Chunhongtangju’ than those in Mandarin ‘Shatangju’ included abscisic acid, 12-hydroxy jasmonic acid, 2-methoxycarbonylphenyl beta-D-glucopyranoside, and ABA-glucosyl ester. ABA has a stronger effect during the maturity of citrus fruits (Romero et al., 2012). The lack of ABA slows the fruit’s ripening process and reduces the sugar content (Zhang et al., 2009; Rook et al., 2010; Forlani et al., 2019). GA3 can slow the fruit’s ripening process, such as tomatoes (Park and Malka, 2022). In this study, gibberellin A24 was enriched in Mandarin ‘Chunhongtangju’ and may perform similar functions to GA3.

### 4.3 Transcriptome profiles of the citrus pericarp at different developmental stages

The development and maturation process of plant fruits is a very complex process, including numerous complex biological processes, such as fruit softening, pigment formation, aroma formation, sugar and acid changes, and flavor formation, which involve the ordered expression of many structural genes and the transcriptional regulatory genes. TFs play a crucial role in regulating various biological processes of plants (Fujisawa et al., 2011; Jin et al., 2014; Shima et al., 2014). The MYB family is involved in plant secondary metabolism, the formation of different organs, pigment synthesis, and transformation, and more (Lea et al., 2007). *StMYB10* regulates the metabolism of the flavonoid during strawberry ripening (Medina-Puche et al., 2014). *MaNACs* can interact with ethylene insensitive 3 (*EIN3*) and contribute to banana fruit ripening (Shan et al., 2012). In the present study, 46 TF families such as AP2/ERF-ERF, MYB, NAC, bHLH, C2C2-Dof, and WRKY were involved in the formation of fruit pericarps during fruit development in two varieties. During the four periods of fruit development, the AP2/ERF-ERF and AUX/IAA families were the top different TF families comparing between two varieties. The down-regulated TF of Mandarin ‘Chunhongtangju’ included AP2/ERF-ERF, AUX/IAA, and MYB. These TFs may have affected the formation of Mandarin ‘Chunhongtangju’ fruit pericarps and need to be further researched.

### 4.4 Differences in expression patterns of genes related to pericarp development

As for the citrus pericarps, the development and ripening process of citrus fruits mainly involves cell division, peel enlargement, chlorophyll degradation, and carotenoid synthesis. The degradation of chlorophyll usually occurs during the senescence of leaves and fruit ripening. *NYC1* gene (Chlorophyll b reductase) and *HCAR* gene (7-hydroxymethylchlorophyll a reductase) can regulate the degradation of chlorophyll (Sakuraba et al., 2013; Teng et al., 2021). During the early development stages of citrus fruit, some color factors, such as lutein and a small number of other carotenoids have started to accumulate. During the maturity process, β-lutein significantly increases, and isomers of lutein and β-cryptoxanthin have been present in large quantities in orange citrus fruits (Katz et al., 2011). *PSY*, *PDS*, and *LCYb1* genes were up-regulated during the maturity. *CsMADS5* contributes to *PSY*, *PDS*, and *LCYb1* genes (Lu et al., 2021). These suggested that the accumulation of carotenoids is regulated by one complex transcriptional network during fruit ripening. There were 2592, and 3587 DEGs involved in the ripening process of Mandarin ‘Shatangju’ and ‘Chunhongtangju’, respectively (**Supplementary Table S5**). In the small and green fruit stage, which is the first stage, the DEGs of CK1 *vs.* LM1 mainly enriched in MAPK signaling pathway-plant, phenylpropanoid biosynthesis, and plant hormone signal transduction such as protein kinase (Cs_ont_8g027870), and anthocyanidin 5-O-glucoside-6’’-O-malonyltransferase (Cs_ont_9g023380); at the second stage, the DEGs of CK2 *vs.* LM2 mainly involved in photosynthesis-antenna proteins, MAPK signaling pathway-plant, biosynthesis of secondary metabolites, and plant hormone signal transduction. Flavin-containing monooxygenase gene (Cs_ont_9g010690), lipoxygenase gene (Cs_ont_2g033670), and ribonuclease H gene (novel.698) were the most significant different genes. When the fruits of ‘Shatangju’ matured, the fruits of ‘Chunhongtangju’ were turning color and not yet ripe, the DEGs were also involved in photosynthesis-antenna proteins metabolic, phenylpropanoid biosynthesis. When the fruits of ‘Chunhongtangju’ matured, the differential expression pattern between the two varieties was mainly reflected in MAPK signaling pathway-plant, photosynthesis-antenna proteins, and phenylpropanoid biosynthesis. Thus, the difference between the two citrus varieties was embodied in energy metabolism, biosynthesis of secondary metabolites, signal transduction, and environmental adaptation.

## 5 Conclusion

In the present study, the fruit development of late-maturing citrus varieties, Mandarin ‘Chunhongtangju’ was significantly slower and the maturity was significantly delayed. At the same period during the ripening process, most hormones in Mandarin ‘Chunhongtangju’ pericarps were higher than those in Mandarin ‘Shatangju’ pericarps such as gibberellin A24, cis-(+)-12-oxophytodienoic acid, and L-phenylalanin. The deficiency of hormones in late-maturing citrus pericarps was mainly manifested in abscisic acid, 12-hydroxy jasmonic acid, 2-methoxycarbonyl phenyl beta-d-glucopyranoside, and ABA-glucosyl ester. Differences in transcription levels of the two citrus varieties mainly show in energy metabolism, biosynthesis of secondary metabolites, and signal transduction such as MAPK signaling pathway-plant, and plant hormone signal transduction. Based on the data of transcriptome and hormones, the top ten genes and hormones that contributed to the result of gene transcription and hormone synthesis included Cs_ont_5g020040, Cs_ont_7g021670, and Cs_ont_2g025760, and 5-Deoxystrigol, Salicylic acid 2-O-β-glucosid, and Gibberellin A24. This work not only revealed the differential mechanism of fruit pericarps between early- and late-maturing citrus varieties but may be of significance in uncovering the unknown gene for molecular breeding.

## Data Availability

The datasets presented in this study can be found in online repositories. The names of the repository/repositories and accession number(s) can be found in the article/**Supplementary Material**.
