# Supplementary material for: Integrated hormone and transcriptome profiles provide insight into the pericarp differential development mechanism between Mandarin ‘Shatangju’ and ‘Chunhongtangju’
Source: Front Plant Sci. 2024 Oct 10;15:1461316. doi: 10.3389/fpls.2024.1461316 (PMC11499144; doi:10.3389/fpls.2024.1461316)
Supplement: Supplementary file 1 [file DataSheet1.docx]

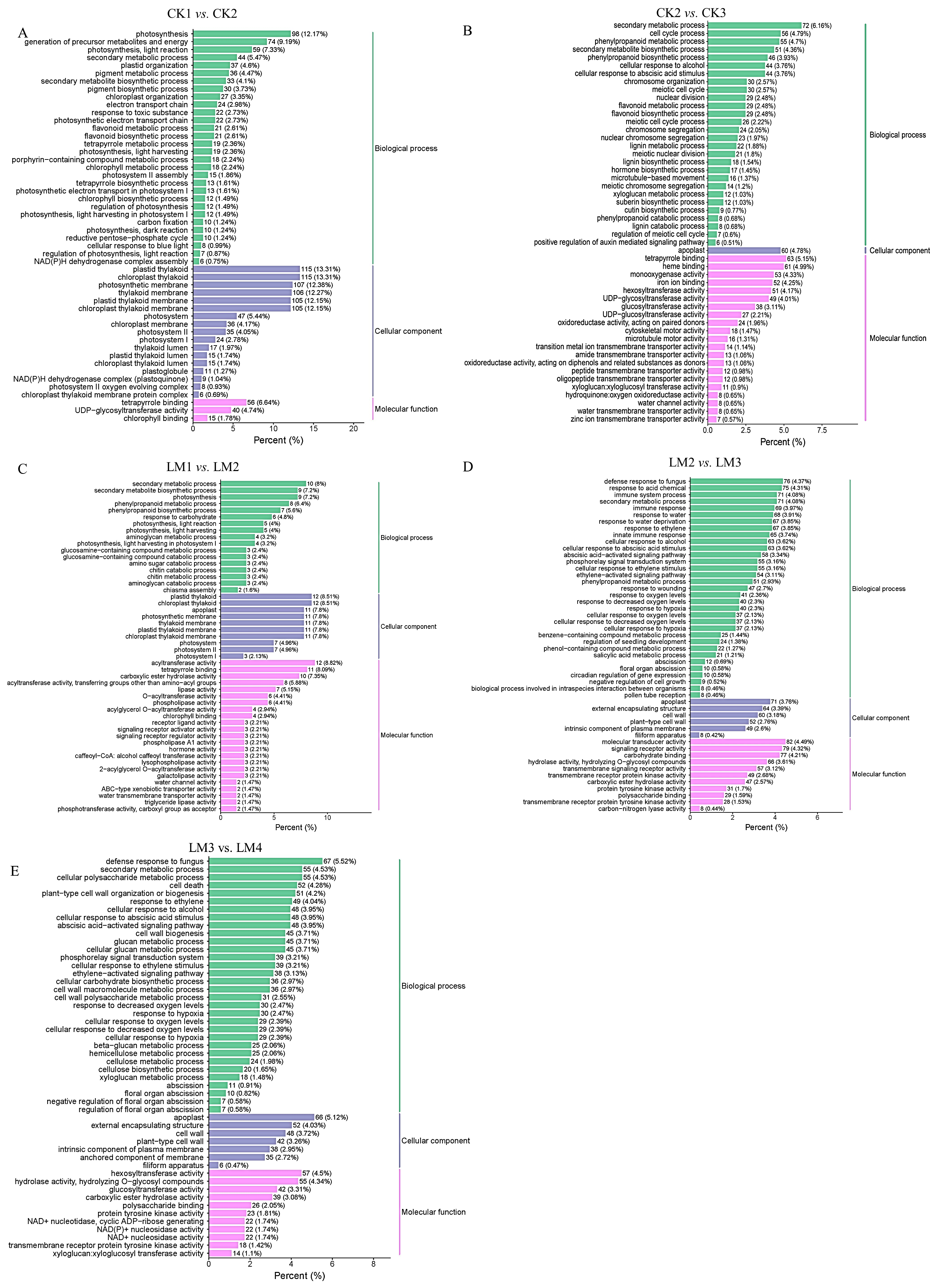


Supplementary Figure S1. Go analysis of DEGs at different development stages


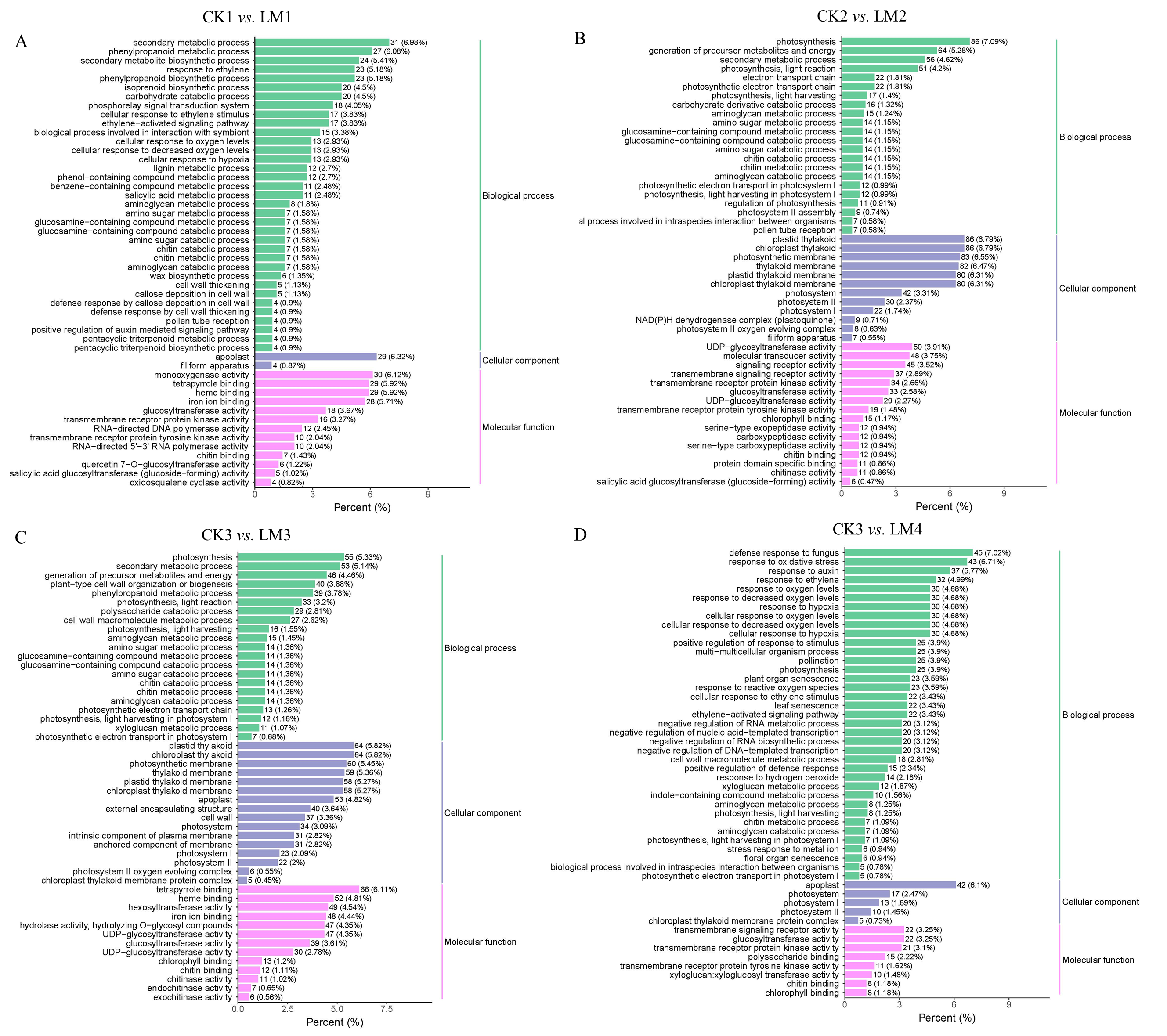


Supplementary Figure S2. Go analysis of DEGs at the same development stages


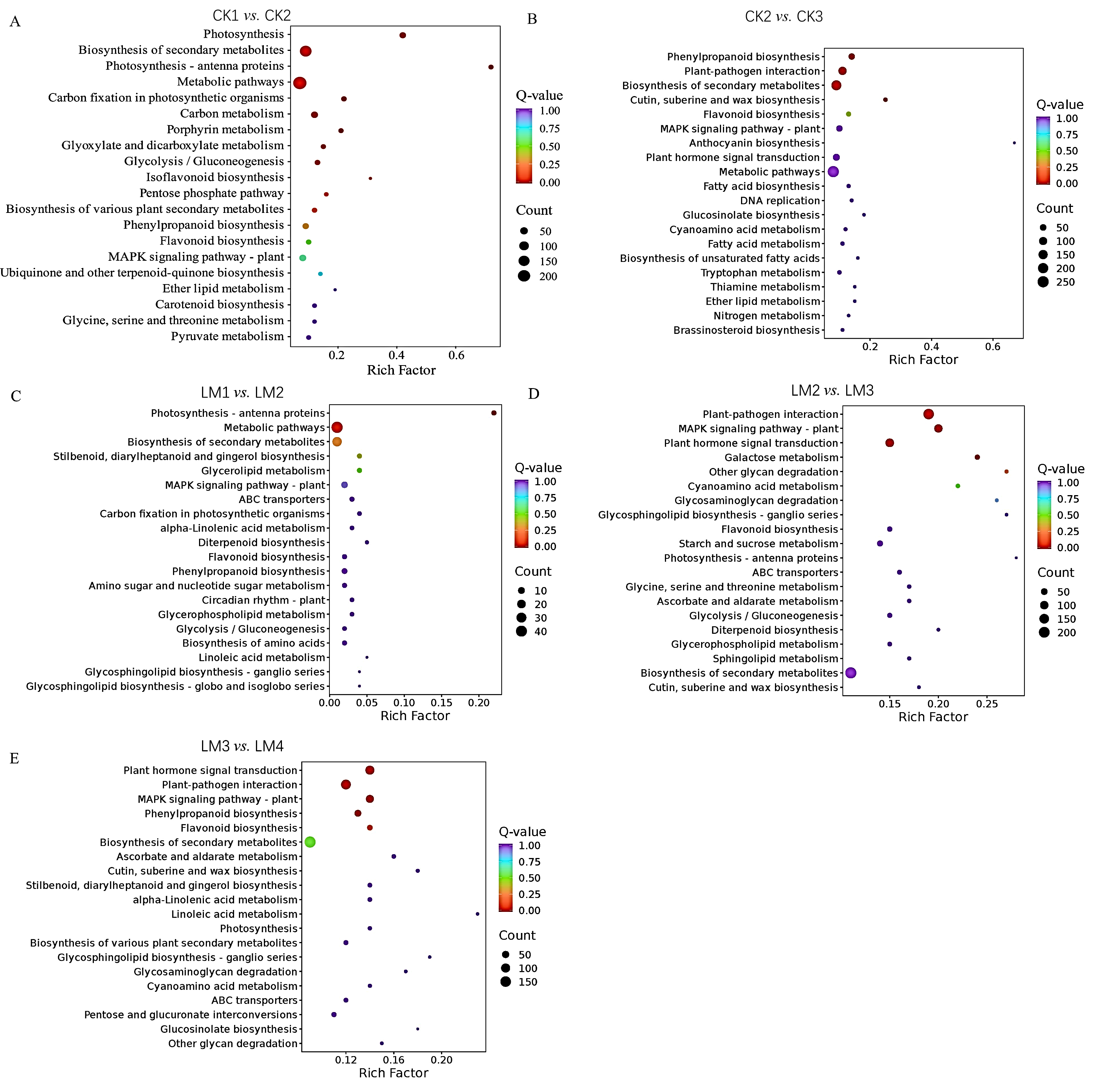


Supplementary Figure S3. KEGG analysis of DEGs at different development stages


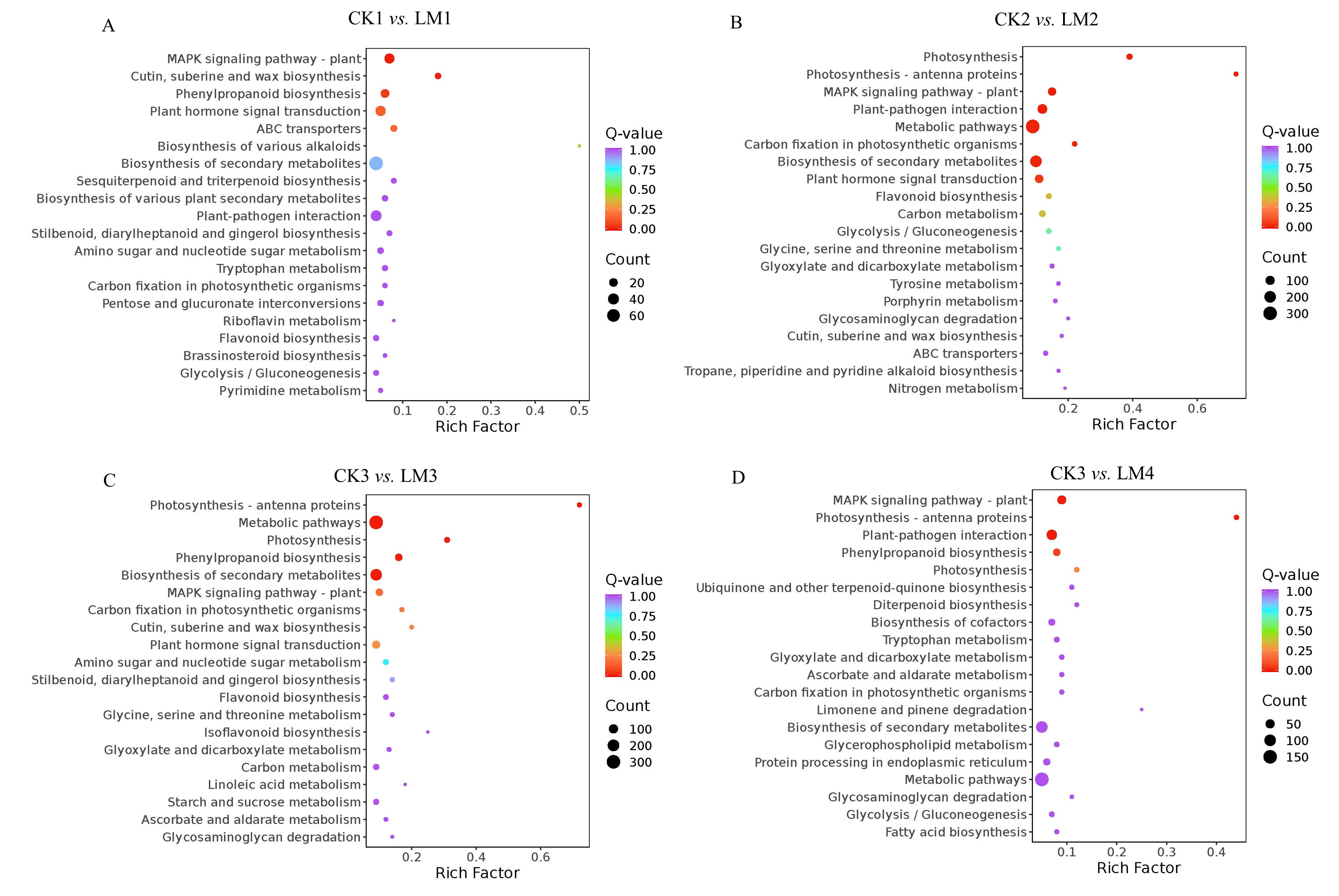


Supplementary Figure S4. KEGG analysis of DEGs at the same development stages

Supplementary Table S11. Primer of ten DEGs for qRT-PCR

| Primer name | Primer sequence |
| --- | --- |
| Cs_ont_5g016050-F | TTGACAATGGCAACAATAACTACTTGG |
| Cs_ont_5g016050-R | GTCGGCGAGTCGGATAATCAATG |
| novel.698-F | GCCGACCTGAAGTTGGAATACAC |
| novel.698-R | CCAGGATTGTGATAGGCGATTCG |
| Cs_ont_3g029910-F | GAGTTGAAGAGATTTCGGTGATTGTG |
| Cs_ont_3g029910-R | GCCTCCAGCCTTGCCTTATTC |
| Cs_ont_2g033670-F | ACTTATTAGTGCTGGTGGAGTCATTG |
| Cs_ont_2g033670-R | CGTCAAATCGCCATTGCTTATCATAG |
| Cs_ont_2g022940-F | GCCGACAGACTGGAAAGAAACC |
| Cs_ont_2g022940-R | ACTCTGCCTTCGTCAACTTCAAC |
| Cs_ont_2g022890-F | GATTCACCTGAGCATTCTGGATGG |
| Cs_ont_2g022890-R | ATCTTGGCAATTAGACAATGAGCAATG |
| Cs_ont_1g017990-F | CCAAAGGAGGCACGAGCAAAC |
| Cs_ont_1g017990-R | ACCAGTTACCTTCAGAATCAGTGTATG |
| Cs_ont_9g010690-F | GCCAACTTCCAAACTTCCCATTTC |
| Cs_ont_9g010690-R | ACTGTTTAGCGTAGGATTCAAGATAGG |
| Cs_ont_5g030100-F | AACAAGCTAGTGGATTACGTCTACAG |
| Cs_ont_5g030100-R | TCCTCCGCTTCCTTCTTCTCTTC |
| Cs_ont_7g002060-F | CTTGCGTCTTCTCTCTTCTCTCAG |
| Cs_ont_7g002060-R | GCGGCCAAATCGAATGTAAACG |
| β-actin-F | CCAAGCAGCATGAAGATCAA |
| β-actin-R | ATCTGCTGGAAGGTGCTGAG |


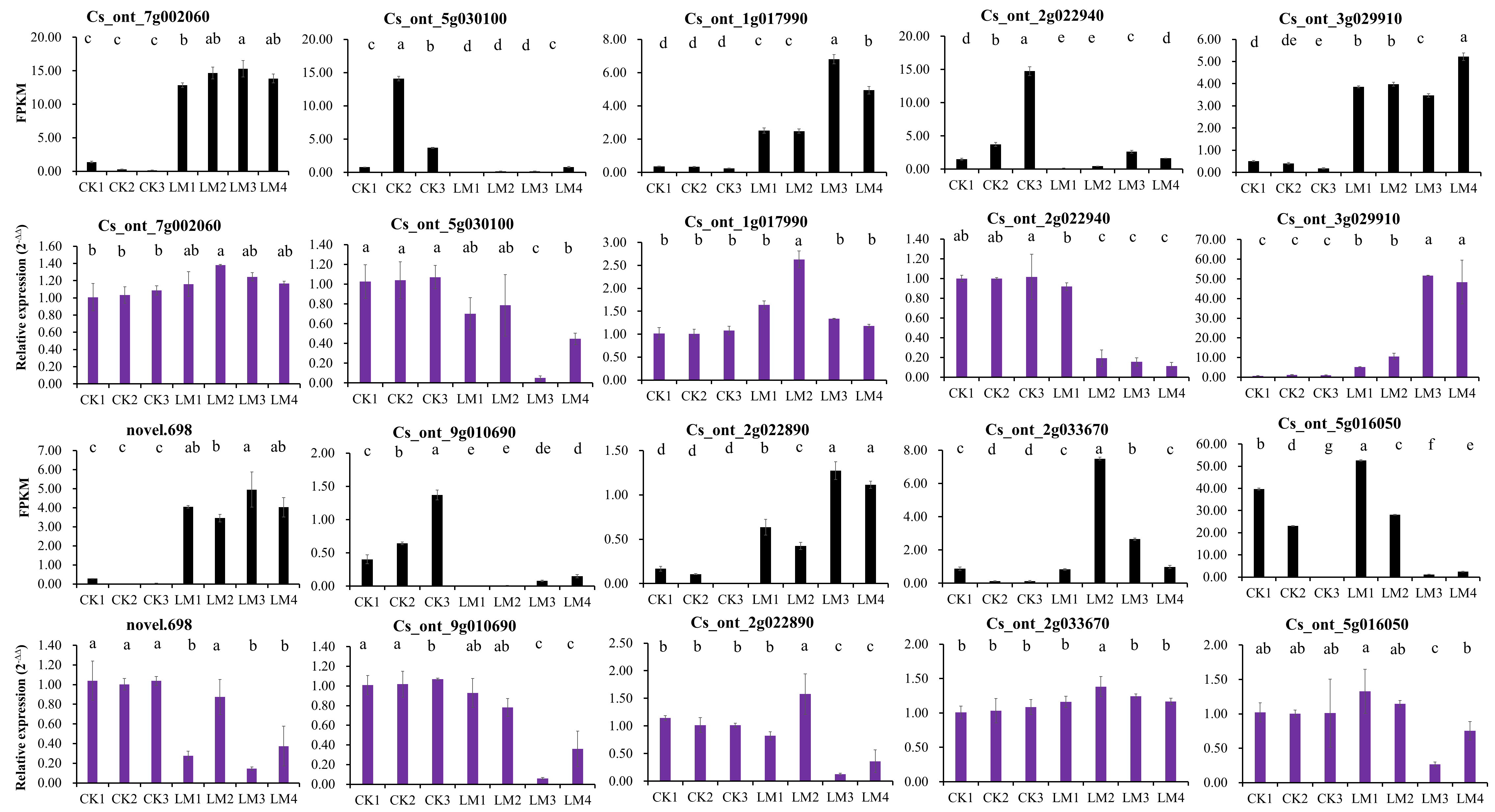


Supplementary Figure S5. qRT-PCR verify the expression levels of ten DEGs


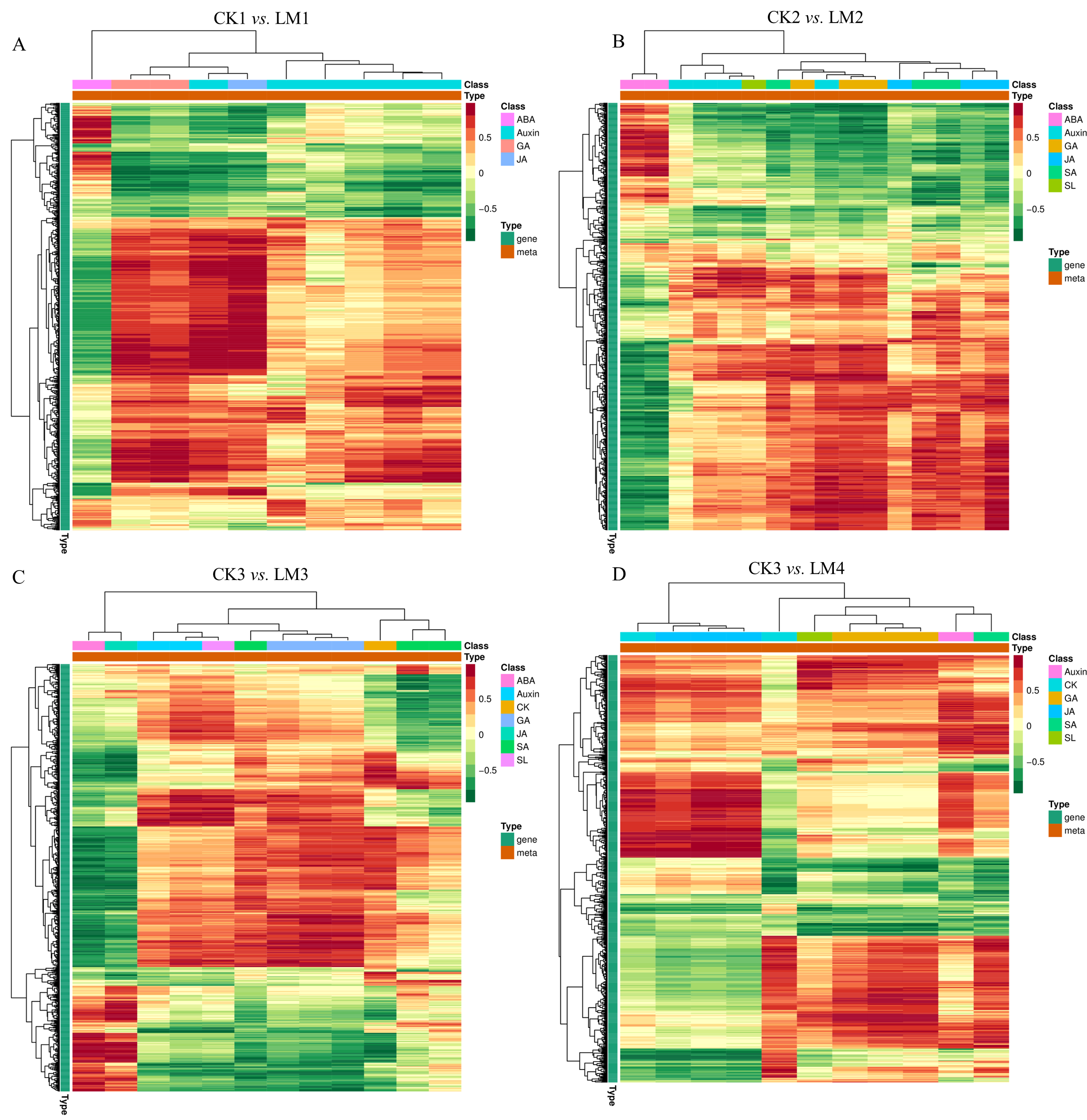


Supplementary Figure S6. Correlation heatmap of DEGs and hormones in the comparison groups. (A) Correlation heatmap of CK1 *vs.* LM1. (B) Correlation heatmap of CK2 *vs.* LM2. (C) Correlation heatmap of CK3 *vs.* LM3. (D) Correlation heatmap of CK3 *vs.* LM4.
